# Evaluating the productivity of ancient Pu’er tea trees (*Camellia sinensis* var. *assamica*): a multivariate modeling approach

**DOI:** 10.1186/s13007-022-00928-5

**Published:** 2022-07-27

**Authors:** Shuqiao Zhang, Wendou Liu, Xinmeng Cheng, Zizhi Wang, Fengjun Yuan, Wengui Wu, Shengxi Liao

**Affiliations:** 1 Institute of Highland Forest Science, Chinese Academy of Forestry, Kunming, 650216 Yunnan China; 2 Fenner School of Environment & Society, College of Science, Australian National University, Canberra, ACT 2601 Australia; 3 Yunnan Institute of Forest Inventory and Planning, Kunming, Yunnan China

**Keywords:** Economical plant resources, Evaluation index, Maximum information entropy, Productivity prediction, Structural equation modeling, Sustainable development

## Abstract

**Background:**

The demand for productive economic plant resources is increasing with the continued growth of the human population. Ancient Pu’er tea trees [*Camellia sinensis* var. *assamica* (J. W. Mast.) Kitam.] are an important ecological resource with high economic value and large interests. The study intends to explore and evaluate critical drivers affecting the species’ productivity, then builds formulas and indexes to make predicting the productivity of such valuable plant resources possible and applicable.

**Results:**

Our analysis identified the ideal values of the seven most important environmental variables and their relative contribution (shown in parentheses) to the distribution of ancient Pu’er tea trees: annual precipitation, ca. 1245 mm (28.73%); min temperature of coldest month, ca. 4.2 °C (18.25%); precipitation of driest quarter, ca. 47.5 mm (14.45%); isothermality, 49.9% to 50.4% (14.11%); precipitation seasonality, ca. 89.2 (6.77%); temperature seasonality, ca. 391 (4.46%); and solar radiation, 12,250 to 13,250 kJ m^−2^ day^−1^ (3.28%). Productivity was indicated by the total value (viz. fresh leaf harvested multiplied by unit price) of each tree. Environmental suitability, tree growth, and management positively affected productivity; regression weights were 0.325, 0.982, and 0.075, respectively. The degree of productivity was classified as follows: > 0.8, “highly productive”; 0.5–0.8, “productive”; 0.3–0.5, “poorly productive”; and < 0.3, “unproductive”. Overall, 53% of the samples were categorized as “poorly productive” or “unproductive”; thus, the management of these regions require attention.

**Conclusions:**

This model improves the accuracy of the predictions of ancient Pu’er tea tree productivity and will aid future analyses of distribution shifts under climate change, as well as the identification of areas suitable for Pu’er tea tree plantations. Our modeling framework provides insights that facilitate the interpretation of abstract concepts and could be applied to other economically valuable plant resources.

## Background

The demand for economically productive plant resources is increasing as the human population continues to grow [1]. Although there have always been trade-offs between economic growth and nature conservation [2], an increase in agricultural productivity (yield) of 60–120% in 2030 relative to 2005 is needed to meet projected increases in demand [3]. Many studies have characterized the relationships of plant productivity with species richness [4] and climate [5], the effects of crown attributes and stand structure on tree productivity [6], and the effects of management policies on plant productivity [7]. Most of these studies have used integrative modeling approaches to explore the relationships among variables [4, 8], but few have evaluated correlations using multi-sourced factors (e.g., environment, plant attributes, and management) at the individual level. In addition, the difficulty of interpreting the significance of the outputs of these modeling analyses often impedes the ability to extract practical insights that could be applied to improve productivity.

Tea is consumed by over three billion people across 160 countries, making it one of the world’s most popular beverages [9, 10]. Its management as a cash crop plays an important role in rural poverty reduction [2, 11] and economic growth. Teas are known to provide benefits to human health and well-being, such as decreasing the risk of cancer [12], cancer recurrence [13], and cardiovascular and nervous system diseases [14]. Rogers et al. [15] demonstrated that an active ingredient in tea, theanine, can have beneficial effects for treating chronic diseases, such as raising blood pressure. The taste of tea can also improve mood and focus and alleviate depression and dementia [16].

China has a long history of tea plantation and culture [17]. Most tea gardens are located in southern China. The Chinese Academy of Agriculture Science classified China into several tea areas based on ecological conditions and geography: South China, Southwest, Jiangnan, and Jiangbei tea areas. Since the economic reform of 1978, the area of tea gardens has increased and reached 0.19 million hectares in 2017, with a total tea yield of 2.46 million tons [18]. Production of the six Chinese traditional tea brands, green tea, yellow tea, white tea, oolong tea, black tea, and dark tea, has grown steadily over the past 40 years, and the tea-based products in the market have diversified. According to Chen et al. [18], China’s tea exports (0.37 million tons in volume) account for 15–20% of the world’s total, and domestic per capita consumption is 1.42 kg per biennium. Yunnan Province, an important region in the Southwest tea area, harbors 15.37% of the tea plantation area and 16% of the total tea output in 2017, making it one of the key areas of strategic development based on tea growing and processing.

Pu’er region, Yunnan Province, is well-known for its high-quality black and dark tea products and unique tea manufacturing process [19], making it an ideal area to study the effect of environmental conditions and human activities on tea production. In Pu’er, many old tea trees found in natural areas are protected. Some trees in tea gardens regrow from the original ancient trunks. According to Lu et al. [9], there is no official definition of ancient tea trees, but natural trees over 100 years of age are often considered ancient. Hence, we define wild trees over 100 years or trees regrown from trunks over 100 years of age as ancient in this study. The main ancient tea species include Dali tea [*Camellia taliensis* (W. W. Sm.) Melch.], tea [*C. sinensis* (L.) Kuntze], Pu’er tea [*C. sinensis* var. *assamica* (J. W. Mast.) Kitam.], and white tea (*C. sinensis* var. *pubilimba* T. L. Ming). Besides values of ecosystem services, ancient teas also have high economic values for their non-timber products. The rich aroma and pleasant taste of these warm dark teas add substantial value to their products [20]. Although the prices of final products depend on fermentation processes, technological infrastructure, and the time required for tea harvesting [21], our field research has indicated that the price of raw teas increases with tree age. Increasing the production of tea from ancient trees could enhance the economic value of these trees, and an analysis of the factors associated with increased productivity is the first step toward achieving this goal.

Here, this study evaluates the productivity of an economically valuable plant resource. To improve the current understanding of interactions between plant productivity and impact factors [1, 3], we detect the critical drivers’ contribution to ancient tea trees’ productivity and build formulas and indexes to help interpretation. We aim to build models that facilitate the objective interpretation of “productivity” and could be applied to other economic plants. The methodology also could be referred to as studying other plant traits and vague concepts.

## Materials and methods

### Study area

Yunnan Province is located in the southern extension of the Qinghai–Tibet Plateau and Yunnan–Guizhou Plateau (from 21° 8′ 32″ to 29° 15′ 8″ N and from 97° 31′ 39″ to 106° 11′ 47″ E). Mountainous regions comprise 84% of the total area of the province, and the plateau and hilly regions account for 10% [22]. Natural plant resources are abundant in Yunnan Province, and the soils are suitable for the growth of various plant resources, as this region (average altitude of 2000 m) is characterized by a unique plateau monsoon climate formed by the South Bengal high-pressure airflow.

**Fig. 1:**
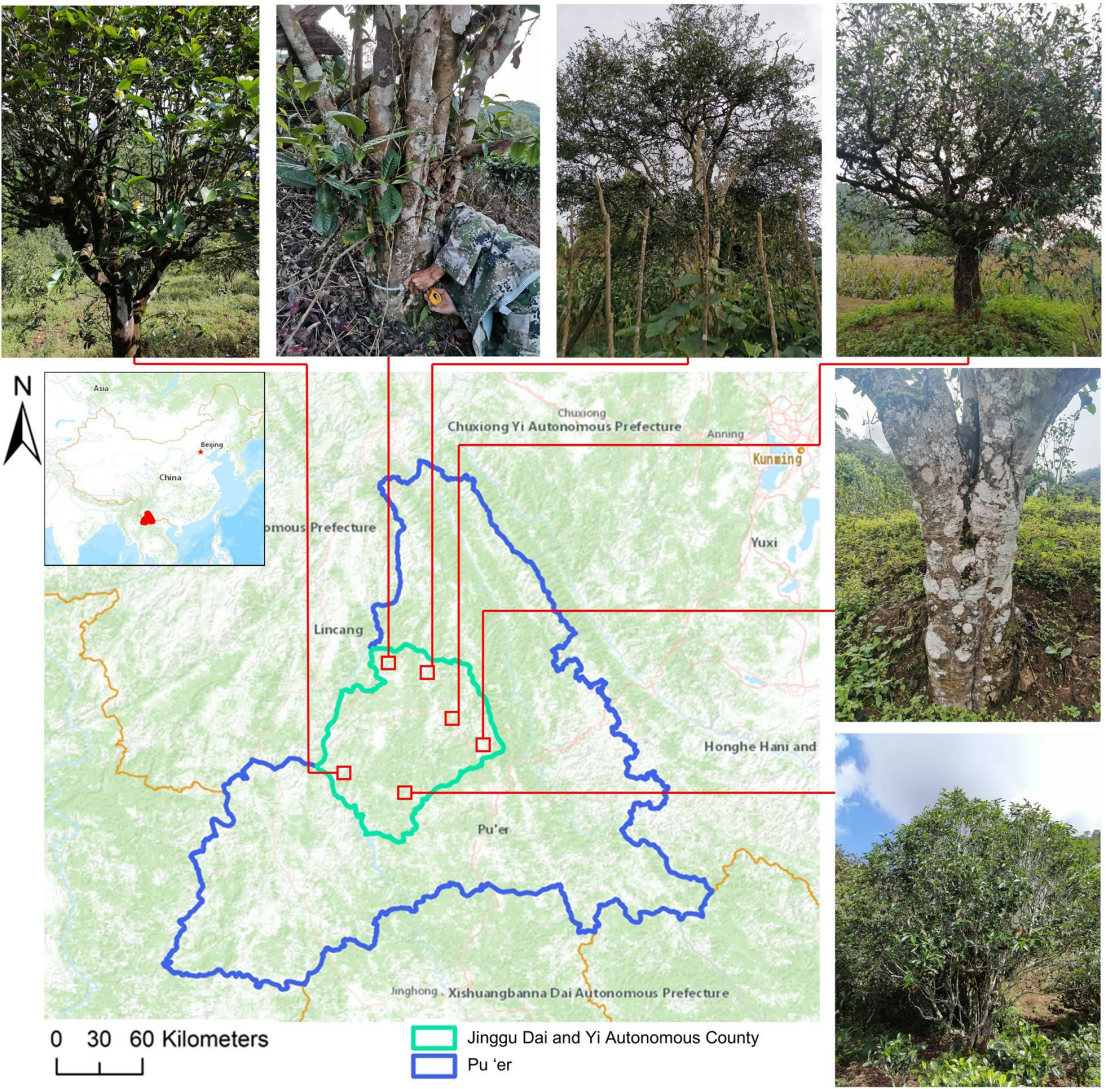
Map of the study area: Jinggu Dai and Yi Autonomous County (light blue), Pu’er Region (dark blue), Yunnan Province, China (orange). Photos are shown of six representative trees

The study area (Fig. 1) is located in southwest Yunnan Province (from 22° 49′ to 23° 52′ N and from 100° 02′ to 101° 07′ E) and covers 777,700 ha. Jinggu Dai and Yi Autonomous County (abbreviated as Jinggu) features a diverse topographic landscape that includes mountainous areas, plateaus, basins, and valleys. The altitude ranges from 600 to 2920 m. The Lancang River flows from northeast to southwest, and there are a total of 94 rivers with a total length of 1863.54 km and annual average total runoff of 4.7 billion m^3^. Jinggu receives abundant rainfall and has distinctive seasons due to the influence of the southwest monsoon. Because Ailao Mountain blocks cold air from the north in winter and monsoon-related precipitation limits increases in temperature in the summer, Jinggu has stable temperatures year-round, but temperature noticeably varies with elevation. The annual average temperature is 17.7 to 22.3 °C [23]. The hottest month (June) features an average temperature of 21.7 to 24.6 °C, whereas the coldest month (January) has an average temperature of 11.4 to 13 °C. The daily and annual temperature differences are 13.6 °C and 11.6 °C on average, respectively. The average annual precipitation is over 1200 mm, and approximately 87% of the precipitation falls during the rainy season (May to October). The average relative humidity is 78%, the average number of annual sunshine hours is 2056.3 h, and southern winds are predominant.

The unique geographical and climate conditions of this region affect the distribution of ancient tea trees. Soil types in the study area are diverse and include red soil, lateritic red soil, yellow soil, brown soil, purple soil, alluvial soil, and paddy soil. Alkali-resistant tea trees grow better on red soil, lateritic red soil, and yellow soil, and these soil types are present across the study area in both valleys and hilly regions. This high diversity of soils has also contributed to the abundance of forest resources. There are 596,000 ha of forestry land, accounting for 79.2% of this region [23]. The dominant forest types are seasonal rain forest, middle mountain moist evergreen broad-leaved forest, Yunnan pine forest, and coniferous and broad-leaved mixed forest. Many ancient tea trees are present in these forest types, and some are protected or cultivated in tea gardens.

### Data

Environmental data collected comprised 28 rasterized parameters (Table 1). The bioclimate variables (bio1–19), wind speed, water vapor pressure, and solar radiation were obtained from WorldClim version 2.1 at a spatial resolution of 30 arc-seconds (1 km^2^). The accuracy of these data was evaluated by global correlation coefficients (between estimated and observed values). All temperature parameters had coefficients over 0.99; solar radiation and vapor pressure had coefficients over 0.95; precipitation variables had coefficients of 0.86; and wind speed had a coefficient of 0.76 [24]. We calculated the average values from the 1970 to the 2000 datasets (released in January 2020). We used public soil texture data from the Resource and Environment Science and Data Center [25], which comprised percentages of sand, silt, and clay. We extracted slope and aspect values from the Advanced Land Observing Satellite (ALOS) 12.5 m Digital Elevation Model (DEM) [26]. To make aspect values more mathematically reasonable, we used sine and cosine functions to constrain the values from − 1 to 1, which represent east–west and north–south degrees, respectively (Table 1).

**Table 1:** Details of the variables, their value ranges, percent contributions to the Maxent model, and permutation importance

Parameter	Full name or description (*)	Value range	Percent contribution	Permutation importance
bio1	Annual mean temperature (°C)	10.29–23.05	0.17	0.33
bio2	Mean diurnal range [mean of monthly (max temp—min temp)] (°C)	9.25–12.95	0.47	1.21
bio3	Isothermality (BIO2/BIO7) (× 100) (%)	47.02–53.25	14.11	6.04
bio4	Temperature seasonality (standard deviation × 100)	307.35–448.04	4.45	5.92
bio5	Max temperature of warmest month (°C)	16.4–36.3	0.01	0.01
bio6	Min temperature of coldest month (°C)	− 2.2 to 11.3	18.25	21.14
bio7	Temperature annual range (BIO5–BIO6) (°C)	18.6–25	0.36	1.71
bio8	Mean temperature of wettest quarter (°C)	14.55–26.45	0.06	0.00
bio9	Mean temperature of driest quarter (°C)	4.87–19.35	0.37	1.15
bio10	Mean temperature of warmest quarter (°C)	14.55–26.45	0.07	0.01
bio11	Mean temperature of coldest quarter (°C)	4.87–17.73	0.34	0.92
bio12	Annual precipitation (mm)	916–1616	29.73	26.75
bio13	Precipitation of wettest month (mm)	173–342	0.12	0.04
bio14	Precipitation of driest month (mm)	5–22	0.04	0.40
bio15	Precipitation seasonality (coefficient of variation)	74.48–90.55	6.77	4.57
bio16	Precipitation of wettest quarter (mm)	486–911	0.01	0.02
bio17	Precipitation of driest quarter (mm)	28–73	14.45	11.97
bio18	Precipitation of warmest quarter (mm)	486–911	0.33	0.65
bio19	Precipitation of coldest quarter (mm)	31–79	1.67	9.78
wind	Wind speed (m s^−1^)	0.7–2.5	0.90	0.55
vapr	Water vapor pressure (kPa)	0.62–1.51	0.21	0.08
srad	Solar radiation (kJ m^−2^ day^−1^)	10,858–14,288	3.28	3.99
sand	*Particle size from 0.05 to 2 mm	22–61	0.13	0.17
silt	*Particle size from 0.002 to 0.05 mm	17–49	0.84	0.96
clay	*Particle size less than 0.002 mm	14–48	0.06	0.07
slope	*Extract from DEM (°)	0–70.49	1.59	0.86
Sin_aspect	*Aspect (east to west) = sin((π/180) × aspect (degree))	− 1 to 1	0.53	0.25
Cos_aspect	*Aspect (north to south) = cos((π/180) × aspect (degree))	− 1 to 1	0.67	0.46

* indicates explanations or essential information of parameters

We measured, monitored, and collected field data from individual ancient tea trees to build a field dataset of ancient tea trees for the past decade. All coordinates of known trees were recorded in Jinggu, which met the “unbiased data” assumption of using Maxent. We obtained comprehensive data from 1282 individuals for the structural equation modeling. Specifically, we measured tree age (years), diameter at breast height (DBH, cm), ground diameter (cm), tree height (m), crown width (m), and height under branch (m). The crown width was the average of the north–south and east–west widths. We also subjectively scored growth vigor (strong = 3, medium = 2, weak = 1), harvest intensity (strong = 1, medium = 0.5, week = 0), and protection (strong = 1, medium = 0.5, weak = 0.1, none = 0). Specifically, trees showing strong morphological growth trends (e.g., crown width and tree height) were considered to show “strong” growth vigor. Harvest intensity was considered “strong” when all new leaves and branches were periodically harvested; by contrast, harvest intensity was considered “weak” when most leaves were had not been removed. Comprehensive protection was considered “strong” if an enclosure was present around the trees, monitoring efforts were in place, and weeds were regularly cleared. These indicators were assessed and recorded in the field. In addition, we summarized each tea tree’s total value based on fieldwork and market research in 2019. The annual production (kg) of green leaf was recorded for each tree by our research group and the local government. Although the final products have different prices due to different processing technology and places it sells, the raw tea from the primary harvesting has nearly the same prices in Yunnan, and the price is related to tree ages [23]. Hence, we used the raw tea’s unit price (RMB/kg) in the study area to calculate the total value by multiplying output (kg).

### Analyses

Here, we evaluated the productivity of tea production from Pu’er ancient tea trees using a structural equation modeling approach. We defined “productivity” as the value generated by an individual tree and used the 1-year output value (viz. fresh leaf harvested multiplied by the unit price) as a direct indicator of productivity. We then developed four models to analyze correlations. The environmental suitability model comprised 28 rasterized parameters. The tree growth model comprised all tree attributes and growth vigor. The management model comprised harvest intensity and protection as variables. The productivity model comprised total value as an indicator.

We used ArcGIS Desktop 10.8 to standardize all environmental raster data into the same band, cell size, pixel type, pixel depth, coordinate system, and spatial datum. We then used Maxent 3.4.4 to calculate contributions from each environmental parameter [27]. Maxent’s maximum information entropy-based machine learning method has been used to filter significant parameters and reduce the number of dimensions [28, 29]. Specifically, we input the geographic coordinates (latitude and longitude) of all known ancient tea trees in Jinggu and 28 rasterized parameters into the software and used the jackknife procedure to measure variable importance. We set the random test percentage as 75, replicates as 10 with cross-validation running type, and the output format as logistic [30]. After running the model, we used the average of 10 iterations to weight the importance of each parameter. We also used the coordinates of 1282 trees to extract values from the 28 rasterized environmental parameters to prepare the dataset for the productivity model using the “Extract Multi values to Points” tool in ArcGIS.

We used JMP 15.2.0 [31] to characterize the distribution of points and detect bivariate relationships between each field parameter and total value. We selected the seven most important environmental variables based on their relative contributions to build the initial integrative model. The seven tree-related and two management-related variables were also comprised. Since the number of parameters is associated with model fitness and redundancy, we tried to filter the most significant variables for the final model by their contributions and rerun the initial model.

We used IBM SPSS AMOS 24.0 to build the integrative model, a graphic-based software to visualize correlations and regression weights among variables [32]. The structural equation modeling method applied in the software has been widely used in recent ecological modeling studies [4, 8]. We built the environment suitability, tree growth, and management models and evaluated their relationships with the productivity model. The initial regressions were calculated based on the following assumptions: environmental suitability affects tree growth, management, and productivity; tree growth affects productivity; and management affects tree growth and productivity (Fig. 4). In addition, we found that fluctuations in total value were correlated with adjustments to the harvest intensity and protection implemented by managers from our fieldwork. Hence, we assumed that the residual errors of harvest intensity and protection were correlated with the residual error of the total value. The final model was standardized by maximum likelihood discrepancy, and unbiased covariances were used as the input matrix. Using the coefficients, we derived mathematical equations to categorize the productivity of our samples into four classes based on the calculated values.

## Results

### Environmental suitability model

**Fig. 2:**
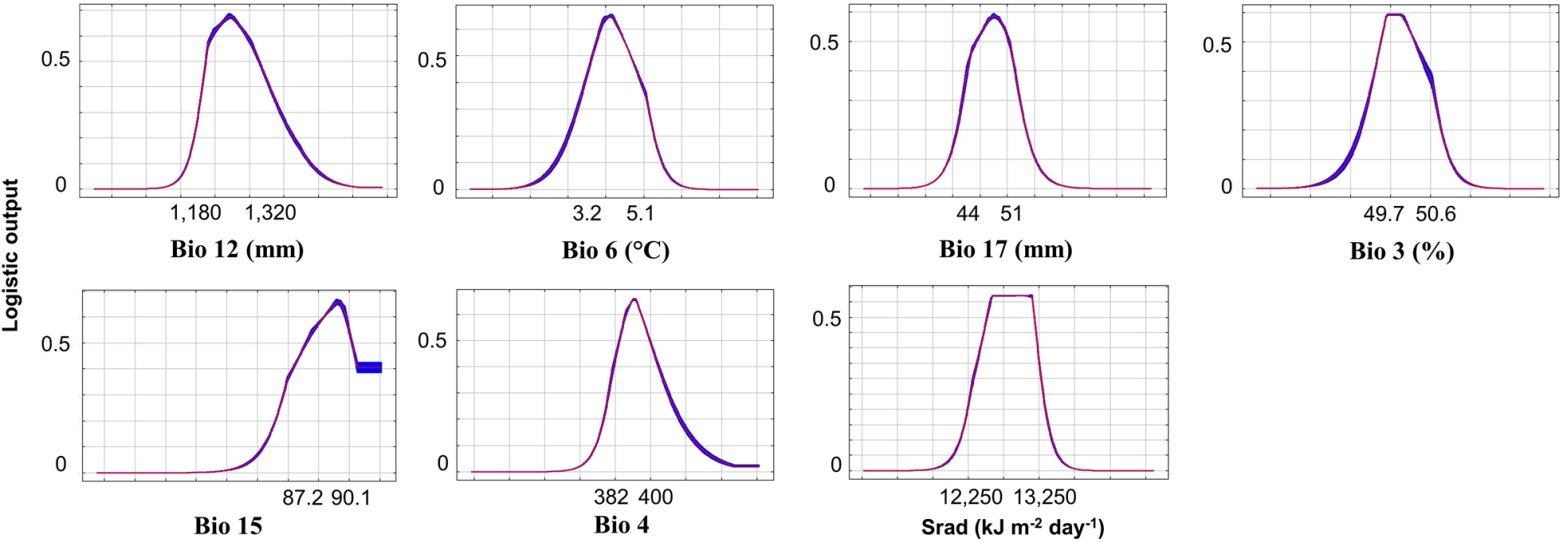
Correlations between each of the top seven variables and the logistic output. These variables are: Bio 12: annual precipitation; Bio 6: min temperature of the coldest month; Bio 17: precipitation of the driest quarter; Bio 3: Isothermality; Bio 15: precipitation seasonality; Bio 4: temperature seasonality; and Srad: solar radiation. Values on the Y-axis indicate the logistic output

The average omission rate over the replicate runs was close to the predicted omission rate per the definition of the cumulative threshold. The area under the receiver operating characteristic curve (AUC) value was 0.938, and the standard deviation was 0.007. The AUC was significantly high for all data partitions, suggesting excellent reliability and fitness of the model [33]. Table 1 shows value ranges, estimates of the relative contributions of the environmental parameters to the Maxent model (all p-values less than 0.01) and parameters’ permutation importance. The seven most-contributed variables were used to build the productivity model and plotted with the logistic output (Fig. 2). These variables are (contribution in parentheses): annual precipitation (29.73%), min temperature of the coldest month (18.25%), precipitation of the driest quarter (14.45%), Isothermality (14.11%), precipitation seasonality (6.77%), temperature seasonality (4.45%), and solar radiation (3.28%). Their curves suggested extreme points that maximize the logistic output, which indicates the effect on environmental suitability. The value 0.5 is a threshold to extract suitable value ranges of parameters, representing suitable environmental conditions of ancient Pu’er tea trees.

### Tree growth and management models

**Fig. 3:**
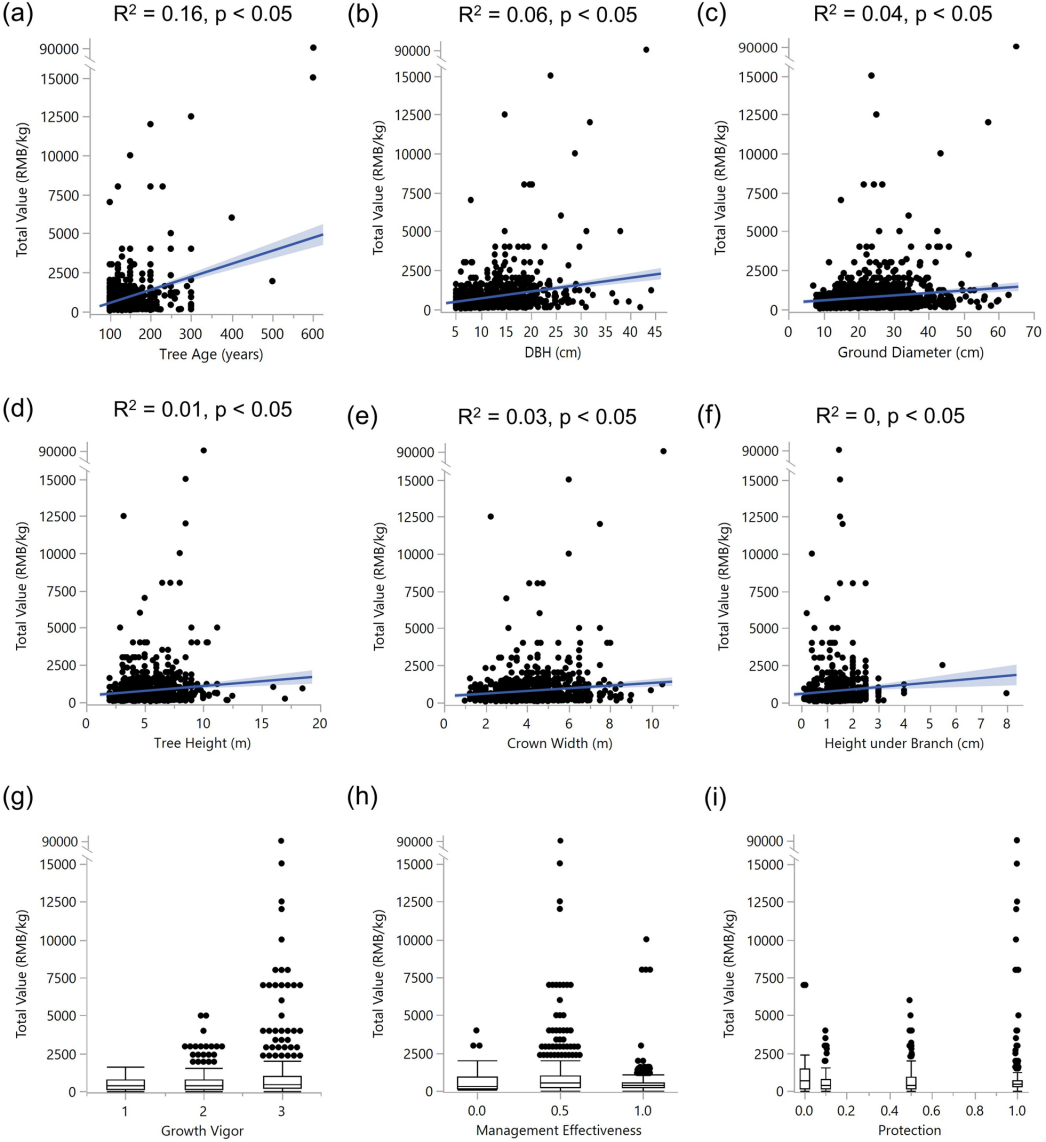
Bivariate relationships of total value (RMB) with six tree growth indicators and box diagrams of quantified evaluating indicators

The bivariate relationships between the total value and tree growth and management-related variables are shown in Fig. 3. We did not observe any clear patterns in these bivariate models but some slightly positive correlations. The results suggest that each bivariate model lack explanatory power with a small R squared. Therefore, integrative pathways are required to explain the relationship between the total value and related variables.

### Productivity model

**Fig. 4:**
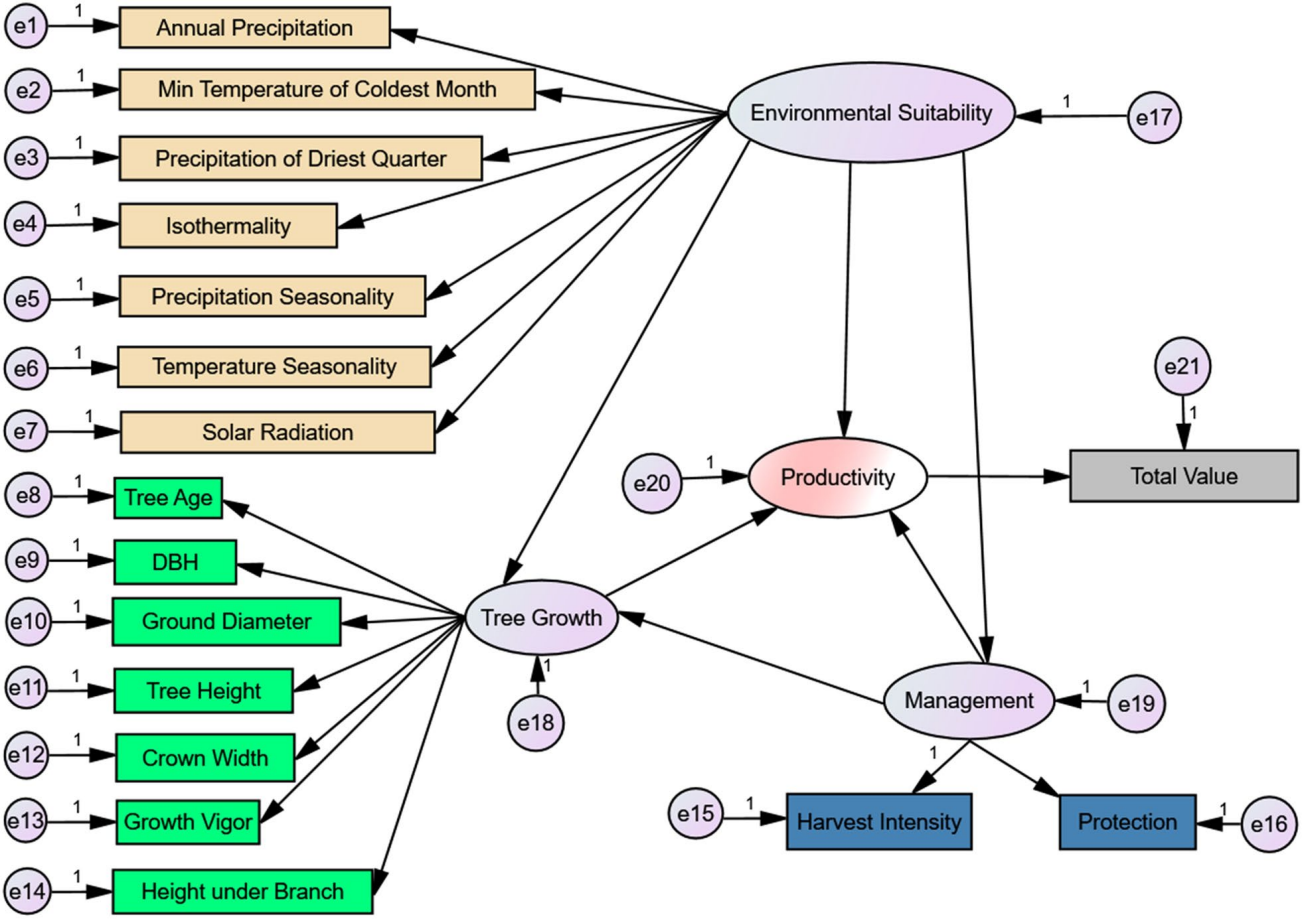
Initial structural equation model with variables and residual errors (e_i_). The variables of the tree growth and management models are our measurements from field investigations. The variables of the environmental model are the seven most contributed ones (Table 1). The variable number (seven) is the same as the variable number of the tree growth model to reduce internal variation. As for the productivity, we used the total value (RMB) of raw tea harvested as an indicator. Correlations connected to residual errors were omitted to aid model visualization. Arrows indicating rectangles from ellipses represent observed variables indicating latent variables. Arrows between ellipses represent correlations

**Fig. 5:**
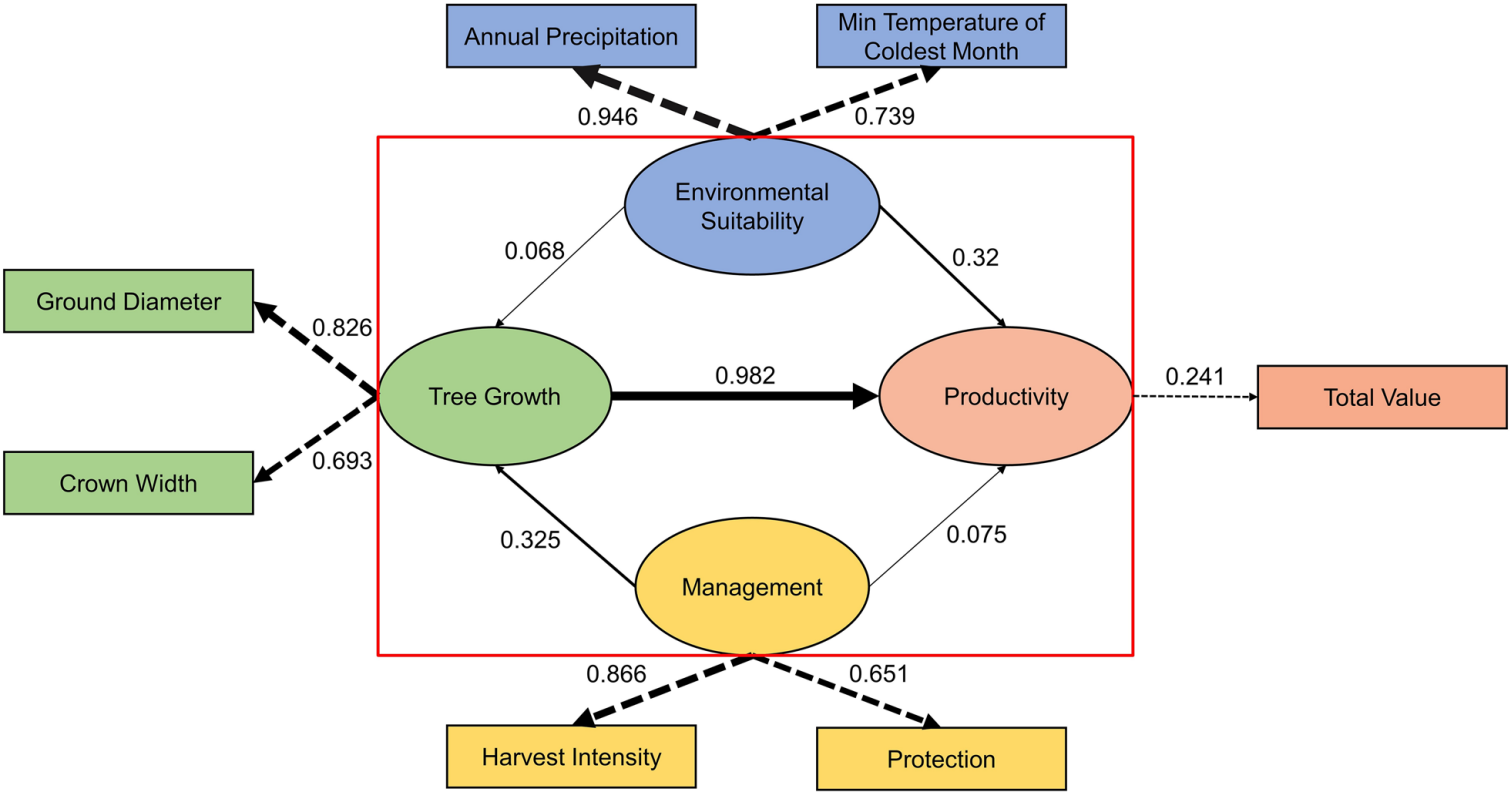
Final structural equation model with key variables and coefficients. Latent variables are drawn in ellipses, and observed variables are drawn in rectangles. Standardized regression weights are shown as solid arrows, and observed variables indicating latent variables are shown as dotted arrows. The coefficient (− 0.619) from environmental suitability to management was not of interest and thus omitted. The red box indicates the relationships among key models. The line thickness indicates the magnitude of the coefficient values. Coefficients next to arrows indicate standardized regression weights

The initial productivity model is shown in Fig. 4. We optimized the model based on variable analysis and their percent contributions to improve model fit. We kept annual precipitation and min temperature of coldest month for the environmental suitability model because they had the highest percent contributions. We left ground diameter and crown width for the tree growth model because they had substantial weights in the initial model. We standardized the units of the parameters by setting residual errors and the magnitudes of some variables to one to make the estimates comparable. The performance of the final model (Fig. 5) was acceptable with the following parameters: chi-square (χ^2^), 34.088; degrees of freedom, eight; goodness of fit index (GFI), 0.993; adjusted GFI, 0.974; comparative fit index (CFI), 0.989; and Akaike Information Criterion (AIC), 74.091.

The results suggest that environmental suitability, tree growth, and management positively contribute to the productivity of ancient tea trees. Because the units were standardized, we used the coefficients (standardized regression weights) to indicate the importance of the parameters: path values indicate variables’ influence on their arrow-pointed models. For example, environmental suitability had a positive but weak effect (coefficient of 0.068) on tree growth, and the effect of environmental suitability was weaker than the effect of management (coefficient of 0.325) on tree growth.

The values of the coefficients suggested that the effect of tree growth on productivity was more than three times the magnitude of the effect of environmental suitability on productivity. The effect of management was very weak. Likewise, the contribution of ground diameter to tree growth was greater than that of crown width, and the effect of harvest intensity on management was greater than the magnitude of the effect of protection on management. The coefficient between total value and productivity was only 0.241, suggesting that there might be other observable parameters (such as residual errors in the model not shown in the Fig. 5) that are better indicators of productivity.

**Fig. 6:**
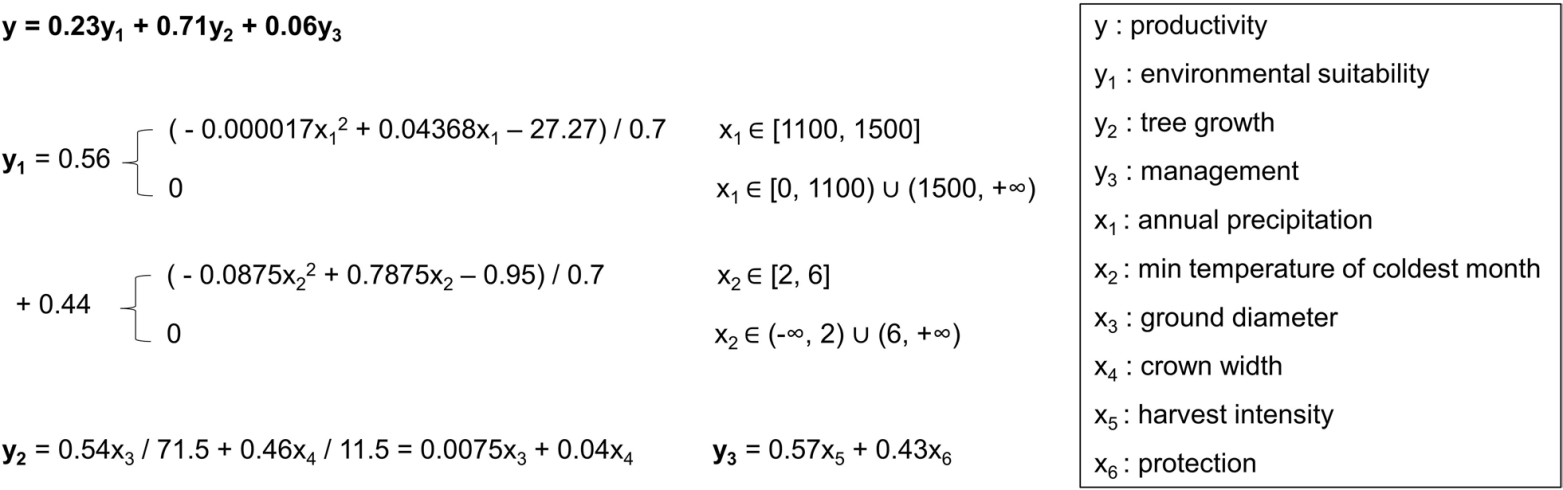
Equations for quantifying productivity (y). Submodel (y_1_, y_2_, and y_3_) weights were determined by coefficients (Fig. 5). The environmental suitability model (y_1_) was formed by quadratic functions based on Fig. 2, and linear models were used for the productivity (y), tree growth (y_2_), and management (y_3_) models

We quantified productivity based on the structural equation model (Fig. 6). The equation was formulated using a linear function by normalizing the coefficients 0.32 (from the environmental suitability model), 0.982 (from the tree growth model), and 0.075 (from the management model) to 0.24, 0.71, and 0.05 (sum is 1), respectively. Likewise, the coefficients of the submodels were determined by the coefficients of the observed variables and standardized by dividing by the maximum values. Specifically, the coefficients 0.54 and 0.46 were determined by the regression weights of 0.826 and 0.693 in the tree growth model, respectively. The denominators 71.5 and 11.5 were the maximum values of the ground diameter and crown width, respectively. The purpose of the division was to standardize their units and make their magnitudes comparable. The coefficients 0.57 and 0.43 were determined by the regression weights of 0.866 and 0.651, respectively. We used quadratic functions to formulate the environmental suitability model (y_1_) because they are the most straightforward pathways for representing the relationships with extrema. The extreme values of parameters (representing the most suitable conditions) and percent contribution (shown in parentheses) to the distribution of ancient Pu’er tea trees are annual precipitation, ca. 1245 mm (28.73%); min temperature of coldest month, ca. 4.2 °C (18.25%); precipitation of driest quarter, ca. 47.5 mm (14.45%); isothermality, 49.9% to 50.4% (14.11%); precipitation seasonality, ca. 89.2 (6.77%); temperature seasonality, ca. 391 (4.46%); and solar radiation, 12,250 to 13,250 kJ m^−2^ day^−1^ (3.28%). The quadratic functions were fit based on these values and Fig. 2. Likewise, their domain definitions were extracted from the function images in Fig. 2; when the logistic output was 0, the environment was not suitable for ancient tea trees, and the factor’s contribution was 0.

**Fig. 7:**
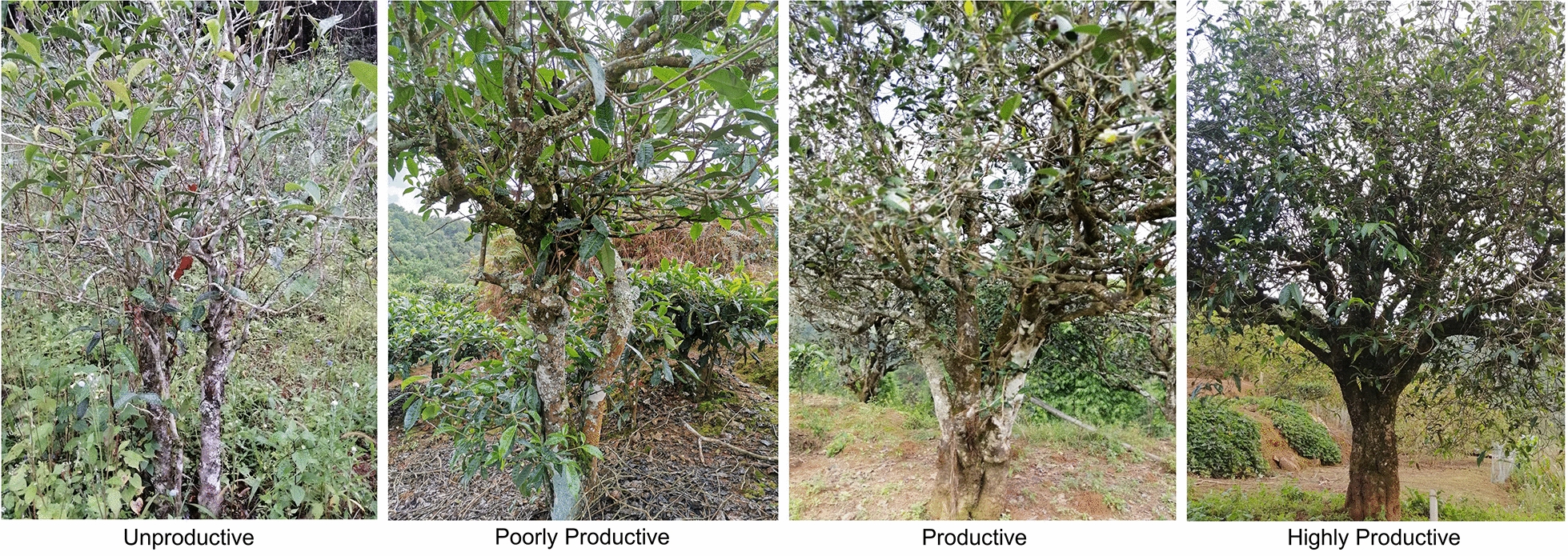
The classification index with representative trees shown. The classification criteria were calculation-based, and experts’ opinions were referred to for assistance

After formulating the model for productivity, we applied the formulas to our samples and set criteria to classify productivity based on the calculations of the equations (Fig. 6), and example photographs of the criteria taken in the field were selected for visual assistance. The criteria were as follows: > 0.8, “highly productive”; 0.5–0.8, “productive”; 0.3–0.5, “poorly productive”; and < 0.3, “unproductive” (Fig. 7). The calculations suggested that 7 samples were highly productive, 594 samples were productive, 667 samples were poorly productive, and 14 samples were unproductive. Overall, 53% of the samples were categorized as “poorly productive” or “unproductive”; thus, these regions’ management requires attention for conservation and productivity improvement.

## Discussion

A comprehensive dataset was obtained in our study. For the environmental suitability model, we comprised nine factors in addition to the commonly used “bio1–bio19” [24] to better reflect environmental factors. The wind speed, solar radiation, and water vapor pressure data were averaged annually instead of quarterly or monthly because ancienttrees have long growth cycles [34]. However, we did not consider whether the extremes (e.g., wind in spring and solar radiation in autumn) limit the distribution of tea trees [35]. Future phytogeographical studies are needed to test this possibility. Although the sprouts and leaves are usually harvested in the spring and autumn, the branches and trunks are slow-growing [36]. Data from the WorldClim database are averaged and interpolated to rasterize the data on maps, which may not be accurate enough for modeling data from individual trees. Our approach could be refined by establishing monitoring points near these ancient tea trees and collecting data regularly, especially in light of the recent impacts of climate change on trees [2].

Among topographic indexes, we used slope and aspect but excluded altitude because we suspected that the effect of altitude on the total value of ancient tea trees would be indirect [37]; we expected that environmental factors such as temperature and precipitation would have direct effects on the total value of ancient tea trees. We used sine and cosine functions to constrain the aspects to the east–west and north–south dimensions (formulas shown in Table 1). Transformed aspect values show better performance in mathematical calculations and theoretically have greater explanatory power. For soils, we used texture because the data were percentages and were easy to calculate. Although this indicator can reflect the soil conditions for tea trees, we did not quantify other important factors, such as types, thickness, and nutrients. These indicators could be used in future studies to better evaluate soil conditions.

The environmental suitability model is a point-based (i.e., individual-based) model. More area-based research is needed to analyze specialized niches, as vegetation structure and landscape components are important in area studies [38]. In future niche analyses, we suggest classifying ancient tea trees into areas consisting of different tea gardens or land types. As Maxent is a coordinate-based method and has been widely used in previous distribution studies because of its simplicity [39], there were studies used random forest or deep learning models to evaluate the contributions of environmental factors [40, 41]. The accuracy and efficiency of the machine learning-based merits comparison in terms of variables’ contribution [42].

The evaluating indicators used in our dataset comprise growth vigor, harvest intensity, and protection. These variables were evaluated in the fieldwork and scored based on our experience. This approach [43] can achieve its intended purpose, but the accuracy and theoretical significance of the characteristics obtained remain questionable. Poor performance of these indicators might be why the management model had a minimal coefficient on productivity. Evaluation of factors such as wildness [44], human activities, and the vegetation community [45] may also provide useful management insights. Although the total value is a straightforward measure for evaluating productivity, other significant indicators could be used in future studies, such as biomass above ground, net primary production, and the dry weight of harvested leaves [46].

The initial structural equation model is comprehensive but shows low fitness and performance because of the high number of variables. The initial model was built using the maximum likelihood technique, and the output parameters were as follows: chi-square (χ^2^), 5987.978; degrees of freedom, 114; all fitness indexes less than 0.7; and AIC, 6065.978. These fitness parameters suggested that the model was unacceptable [32]. Thus, we retained the top two most important variables for the environmental suitability model. For the tree growth model, we omitted the other five parameters for the following reasons aside from their lower regression weights [23]: (1) tree age: numbers are reported by locals and are often rough estimates (more robust ways to measure it are required in the future, such as isotope tracing methods [47] and dendrochronology methods [48]); (2) DBH: most trunks bifurcate below breast height; (3) tree height: some managers artificially limit heights to promote the growth of well-developed canopies [23]; (4) growth vigor: it is quantified in only three classes and thus cannot clearly separate individuals; and (5) height under branch: it determines intervals for intercropping and thus does not strongly reflect tree growth conditions [49].

In quantifying productivity, we noticed that the curves (Fig. 2) between environmental suitability variables and logistic output have extreme points. This is reasonable given that every plant species has preferences for specific climate factors such as temperature and precipitation [50]. We used quadratic functions with different domain definitions to fit the relationships, given that we sought to minimize the model’s redundancy. For this same reason, we used linear functions to formulate the productivity, tree growth, and management models. Simplified functions are efficient for evaluating data from many individuals. Regarding the criteria-based indexes, we categorized ancient tea trees into four levels based on outputs from the formulas (Fig. 6), our field evaluations, and local reports [23].

Bivariate relationships in the tree growth model (Fig. 3) were lacking in explanatory power for various possible reasons. First, the correlations between tree parameters and productivity are indirect. Because we used the total value to indicate productivity, the direct parameters and harvested tea parts (such as sprouts and leaves) are related. Although parameters such as crown width can reflect tree growth conditions and theoretically represent the production of trees, their linear relationships with total values might be weak. Another reason might be that we included many ancient tea trees varying in age in the study area. The tea of some trees over 500 years old may have remarkable prices but low output. Their size-related growth parameters are usually high compared with younger plants. However, young trees having lower growth parameters may have high output and low prices. In this case, their total values are close, but growth differs greatly. The large data size and skewed data distribution may also result in non-significant model fit for some parameters. We did not apply any transformations in terms of specific bivariate relationships because the original data are more logistic in explaining correlations in the final model, and we wanted to ensure that the entire dataset satisfied a multivariate normal distribution.

The significance of our model is that productivity can be determined based on measures from environments, trees, and management evaluations without harvesting the leaves of ancient tea trees. Managers can gain insight and make decisions by classifying trees into categories before harvesting. For example, the term “poorly productive” is more easily interpreted than the term “low net primary production” or “small biomass.” “Highly productive” and “productive” trees can be harvested twice a year. “Poorly productive” trees can be harvested once a year. “Unproductive” trees should not be harvested to promote their healthy growth. However, we only used data from one county to build the formula and index. An analysis includes other regions with ancient tea trees distributed will greatly improve the robustness of the evaluation index. Our productivity model of ancient tea trees could also potentially be applied to other economically important tree plants, such as lacquer, fruit, and coffee trees.

The structural equation modeling approach for studying productivity is effective for exploring the significance of unobserved variables [51]. Quantitative modeling can facilitate the interpretation of abstract concepts. The indexes can aid interpretation of the practical significance of model outputs, which can then be used to modify management approaches accordingly. This approach can also be used to clarify other abstract concepts. For example, the heights and growth forms of trees have been quantified and then evaluated in indexed classes such as the Australian National Vegetation Information System [43].

Ancient tea trees are different from other ancient tree resources because of their high economic value and sustainable management models. Unlike other economically important tree species with unsustainable timber products, for example, precious wood [52], tea products are produced periodically. Hence, their conservation and sustainable production require special attention. A quantitative modeling approach for evaluating productivity represents a first step in the study of ancient tea trees. Several outstanding research questions remain. For example, in addition to their economic and ecological significance, what are some of the political, societal, and cultural implications of ancient tea trees? How generalizable is the productivity model beyond ancient Pu’er tea trees? Are there differences between wild and cultivated ancient tea trees in terms of their productivity? Can Pu’er tea species be cultivated in other regions? Might it be possible to develop technology that allows normal tea products to possess the same tastes and scents of tea derived from ancient Pu’er tea trees? Can the production of ancient teas be increased sustainably? Such questions will require additional research.

In subsequent research, we plan to model the niches and distributions of ancient Pu’er tea species under future scenarios of climate change and globalization. We also plan to explore potentially suitable areas for Pu’er tea tree plantations in regions with tea cultures and plantation interests. Practical management actions need to be implemented in different regions and should be based on current management methodologies.

## Conclusion

In this study, we used a structural equation modeling approach to quantify the productivity of tea production from ancient Pu’er tea trees and employed various indexes to permit qualitative evaluation. Overall, the model exhibited acceptable performance and permitted the identification of significant variables to include in the submodels. The final model suggested that environmental suitability, tree growth, and management positively affected productivity; regression weights were 0.32, 0.982, and 0.075, respectively. The index suggested 53% of the samples require management attention. In addition, our environmental suitability submodel revealed the optimal environmental conditions for ancient Pu’er tea trees. Modeling productivity is the first step to achieving the sustainable management and growing of Pu’er tea in potential interested regions. Future studies are needed to analyze specialized niches and distribution patterns under future climate change and the plausibility of establishing plantations in other areas.

The quantitative model and qualitative index provide a more robust approach for quantifying productivity compared with traditional correlation-based approaches. They also contribute to the local managers’ interpretation and prediction of the trees’ production before harvesting. In addition, the methodology might be applicable beyond ancient Pu’er tea trees (e.g., other economically valuable trees and plant resources requiring conservation attention) and could provide insights that facilitate the interpretation of abstract concepts, but its generality requires further examination.

## Data Availability

The datasets generated during and/or analyzed during the current study are available from the corresponding author on reasonable request.
